# Characteristics, utilisation and influence of viewpoint articles from the Structured Operational Research and Training Initiative (SORT IT) – 2009-2020

**DOI:** 10.12688/f1000research.27349.1

**Published:** 2021-03-10

**Authors:** Mohammed Khogali, Katie Tayler-Smith, Anthony D. Harries, Rony Zachariah, Ajay Kumar, Hayk Davtyan, Srinath Satyanarayana, Olga Denisiuk, Johan van Griensven, Anthony Reid, Saw Saw, Selma Dar Berger, Veerle Hermans, Abraham Aseffa, John C. Reeder

**Affiliations:** 1 Research for Implementation , UNICEF/UNDP/World Bank/WHO Special Programme for Research and Training in Tropical Diseases (TDR), World Health Organization, Geneva, Switzerland; 2 LuxOR, Médecins Sans Frontières, Operational Centre Brussels, Luxembourg, Luxembourg; 3 Centre for Operational Research, International Union Against TB and Lung Disease, Paris, France; 4 London School of Hygiene and Tropical Medicine, London, UK; 5 International Union Against Tuberculosis and Lung Disease, New Delhi, India; 6 Yenepoya Medical College, Mangalore, India; 7 Tuberculosis and Research Center NGO (TB-RPC), Yerevan, Armenia; 8 Alliance for Public Health, Kyiv, Ukraine; 9 Institute of Tropical Medicine, Antwerp, Belgium; 10 Department of Medical Research, Ministry of Health and Sports, Pyin Oo Lwin, Myanmar

**Keywords:** viewpoints, utilization, SORT IT, policy and practice

## Abstract

**Background**: The Structured Operational Research and Training Initiative (SORT IT) teaches the practical skills of conducting and publishing operational research (OR) to influence health policy and/or practice. In addition to original research articles, viewpoint articles are also produced and published as secondary outputs of SORT IT courses. We assessed the characteristics, use and influence of viewpoint articles derived from all SORT IT courses.

**Methods: **This was a cross-sectional study involving all published viewpoint articles derived from the SORT IT courses held from August 2009 - March 2020. Characteristics of these papers were sourced from the papers themselves and from SORT-IT members involved in writing the papers. Data on use were sourced from the metrics provided on the online publishing platforms and from Google Scholar. Influence on policy and practice was self-assessed by the authors of the papers and was performed only for papers deemed to be ‘calls for action’.

**Results: **A total of 41 viewpoint papers were published. Of these, 15 (37%) were ‘calls for action’. In total, 31 (76%) were published in open-access journals and the remaining 10 in delayed access journals. In 12 (29%) of the papers, first authors were from low and middle-income countries (LMICs). Female authors (54%) were included in 22, but only four (10%) and two (5%) of first and last authors respectively, were female. Only seven (17%) papers had available data regarding online views and downloads. The median citation score for the papers was four (IQR 1-9). Of the 15 ‘call for action’ papers, six influenced OR capacity building, two influenced policy and practice, and three influenced both OR capacity building within SORT IT and policy and practice.

**Conclusion: **Viewpoint articles generated during SORT IT courses appear to complement original OR studies and are valued contributors to the dissemination of OR practices in LMICs.

## Background

In 2009, the International Union Against Tuberculosis and Lung Disease (The Union) and Médecins Sans Frontières (MSF) began an operational research (OR) training course. In 2012, the Special Programme for Research and Training in Tropical Diseases (TDR), hosted at the World Health Organisation (WHO), adopted this model and formed a partnership-based programme known as the Structured Operational Research and Training Initiative (SORT IT). SORT IT seeks to strengthen health systems, improve programme performance, and enhance public health through OR. To this effect, SORT IT enrols participants from low and middle-income countries (LMIC) and teaches them the practical skills of conducting and publishing OR in peer reviewed scientific journals with the goal of influencing policy and/or practice ^1^. Details of the structure and organisation of the SORT IT programme have been described elsewhere ^1^.

In addition to capacity building, each SORT IT course brings together a rich diversity of people (faculty and participants) from different backgrounds, expertise, experience and countries. This richness often gives rise to vibrant discussions, new ideas and innovative reflections on health-related issues of global interest. Various aspects of OR capacity building to improve public health are also discussed and critically reflected upon. This dynamic - which characterises the *modus-operandi* of SORT IT – has resulted in the conception and publication of over 40 “viewpoint” articles (classified by journals as viewpoints, editorials, and perspective articles). These viewpoint papers are secondary outputs of SORT IT courses and present critical analysis of a health or policy issue of interest ^2^ and/or challenge the norms of practice (“business as usual”). Many of these papers include a ‘call for action’ to improve public health or advocate for effective approaches to OR capacity building.

Since the inception of SORT IT, we have closely monitored and evaluated the characteristics and outputs of the OR courses, together with the influence that original research papers have had on policy and practice ^3– 9^. However, we have yet to carry out any formal assessments of viewpoint articles – in particular examining who are the voices behind the papers, to what extent the papers have been used by others and whether/how they might have influenced policy and or practice. 

This study aimed to assess the characteristics, utilisation and influence of published viewpoint articles derived from all SORT IT courses conducted between August 2009 and March 2020. Specific objectives were to report on i) the characteristics of publications, including LMIC contributions to authorship, collaborative partnerships (all High Income Countries - HIC vs HIC-LMIC); ii) access type and use of published evidence (views, downloads and citations); and iii) how ‘call for action’ papers influenced the implementation of OR capacity building within the SORT IT partnership, and policy and/or practice at national and international levels.

## Methods

### Design

This was a cross-sectional study involving all published viewpoint articles derived from SORT IT courses conducted between August 2009 and March 2020. Given that journals, publishers and academic communities do not always use standardised terminology for perspective and opinion pieces, we have decided to use the term ‘viewpoint article’ in this study. It includes any publication outside the categories of original research or reviews that discusses a relevant topic from the perspective of the author(s).

### SORT IT courses and publications

Between August 2009 and March 2020, there were 86 SORT IT courses conducted in Asia, Africa, Europe, South Pacific, and Latin America. A full description of the SORT IT courses, including criteria for selection of participants, programme structure and milestones, mentorship and facilitators has been described previously ^1^. In brief, a regular SORT-IT course consists of three one-week modules implemented over a period of 10 to 12 months. During Modules 1 and 2, participants develop research protocols and electronic data capture and analysis tools. Module 3 focuses on writing a manuscript for scientific publication ^10^. Specific milestones must be achieved if participants are to proceed from one module to the next. A participant is considered to have successfully completed the course if they complete all milestones, including submission of a final manuscript to a peer-reviewed journal. Up until March 2020, almost all modules were residential, during which participants and facilitators would gather every day for five-six days. Diverse discussions related to global health issues and OR capacity building took place during the courses and these served as the driving force behind the viewpoint papers that originated from these SORT IT courses.

### Data collection and analysis

In June 2020, viewpoint articles were obtained from a dedicated database (see underlying data ^13^) that contains all SORT IT viewpoint article publications. Information on the characteristics of the viewpoint papers were sourced from the papers themselves and through consultation with authors. Variables included type of paper (Call for action – papers requesting a change in policy or practice; Descriptive – papers describing an OR or public health related issue), number of authors, nationality, gender and institutional affiliation of the authors, and whether they were involved with work in LMIC.

Data pertaining to journal access (open, delayed or subscription-based access) were obtained from each journal’s website; data on a paper’s views and downloads were retrieved from metrics provided by the online publishing platform indexing the article; citation counts were ascertained by examining and cross-checking the citations listed (for each given paper) on the online publishing platform and also in Google Scholar. Final citation counts for each paper were obtained by counting the citations common to both lists, together with any citations found only on the online publishing platform and/or only in Google Scholar.

Influence on policy and practice was only assessed for papers deemed to be ‘calls for action’. Each paper was examined by a committee of its senior authors in June 2020, allowing time for more recent call for action papers to potentially influence policy and practice (papers having been published between 2010 and 2019). A discussion took place about whether international / national guidelines were modified or changes in policy and/or practice were observed at international or national level as a result of the publication.

Data related to the study objectives were collected and analysed during June 2020.

Data were entered into Microsoft Excel 2016. Frequencies and proportions were used to summarise categorical variables, and medians and interquartile ranges were used to report continuous variables.

## Results

### Characteristics of the viewpoint articles

As a result of the SORT-IT courses held between August 2009 and March 2020, a total of 41 viewpoint papers were published; their characteristics are shown in Table 1. Just over a third of the papers constituted ‘calls for action’ (15, 37%). Fewer than a third of first authors were from an LMIC (12, 29%); however, the majority of first authors were involved in work in LMIC (40, 98%) and over two thirds of the papers included authors from LMICs (29, 71%). Only 54% of papers included female authors, and only 10% and 5% of first and last authors, respectively, were female.

**Table 1.** Characteristics of viewpoint papers resulting from SORT IT courses, August 2009 – March 2020 (n-41).

	n (%)
**Type of paper** Call for Action Descriptive	15 (37) 26 (63)
**Median number of authors per paper (IQR) [range]**	8 (4 – 12), [2 –35]
**Origin of authors** *First author* LMIC HIC *Last author* LMIC HIC *All authors* LMICs only HICs only LMICs and HICs	12 (29) 29 (71) 6 (15) 35 (85) 1 (2) 12 (29) 28 (68)
**First author involved in LMIC work** Yes No	40 (98) 1 (2)
**Gender of authors** *First author* Male Female *Last author* Male Female *All authors* Male only Female only Male and female	37 (90) 4 (10) 39 (95) 2 (5) 19 (46) 0 22 (54)
**Type of institutional affiliations represented by authors** NGO Academic institute MoH WHO	35 (85) 38 (93) 19 (46) 16 (39)
**Type of institutional collaboration** LMIC-LMIC ^1^ HIC-HIC ^2^ HIC-LMIC ^3^	0 (0) 11 (27) 30 (73)

LMIC, Low-middle income country; HIC, High income country; IQR, Interquartile range; NGO, Non-governmental organisation; MoH, Ministry of Health; WHO, World Health Organisation.

^1^ All author institutions represented on the papers from LMIC

^2 ^All author institutions represented on the papers from HIC

^3^ Some author institutions from the HIC and some from LMIC

Most of the papers included NGO (35, 85%) and academic (38, 93%) author affiliations; 19 (46%) included a ministry of health affiliation and 16 (39%) included a WHO affiliation (some authors had more than one affiliation and these were included in the analysis). There was HIC-LMIC institutional collaboration in over two thirds of the papers (30, 73%). 

### Access to and utilisation of the viewpoint papers

Of the 41 viewpoint papers, 31 (76%) were published in open access journals and the remaining 10 (24%) in delayed access journals. Online data for article views and downloads were only available for seven (17%) of the papers published in the following journals: BMC Research Notes (2), Frontiers of Public Health (1), Pan American Journal of Public Health (1), Global Health Action (1), F1000 Research (1) and BMJ Global Health (1). The paper with the highest number of views and PDF downloads (3322 and 1089) was a ‘call for action’ paper published in 2019 ( see underlying data ^13^). An overview of citation counts for all papers is shown in Table 2. The median citation score for the call for action papers was four (IQR 2-11) and thus was no different to the median citation score for the descriptive papers (4, IQR 1-8) (data not shown in Table).

**Table 2.** An overview of citations resulting from SORT IT viewpoint papers ^1^ according to their year of publication.

	No. of papers (n)	Citations ^2^	
		*Median*	*IQR*
**Total**	41	4	1 – 9
**Publication year of Discussion paper** 2010 & 2011 2012 & 2013 2014 & 2015 2016 & 2017 2018 & 2019	2 12 13 6 8	32 8 5 2 1	26 – 37 4 – 24 3 – 7 1 – 4 0 – 1

IQR, Interquartile range

^1^ Up until June 23 ^rd^, 2020

^2^ Total number of unique article citations based on the results shown on the online publishing platform and in Google Scholar (For 18 of the papers, Google Scholar retrieved more article citations than the online publishing platform)

### Influence on OR capacity building and on policy and/or practice

Influence on OR capacity building within the SORT IT partnership and on policy and practice was assessed for the 15 papers that constituted a ‘call for action’. Table 3 provides an overview of these papers and the reported influence. Of these, 11 (73%) resulted in some form of change: six papers influenced the evolution of OR capacity building within the SORT IT partnership, two influenced policy and practice at the national or international level, and three influenced both OR capacity building within SORT IT and policy and practice.

**Table 3.** Influence of SORT IT ‘call for action’ papers on OR capacity building and on national and international policy/practice (n-15).

Title of paper	Influence of the call for action papers ^*^		
	None	OR capacity building within SORT IT	National and International policy and practice
The published research paper: is it an important indicator of successful OR at programme level?		√	
Language in tuberculosis services: can we change to patient-centred terminology and stop the paradigm of blaming patients?			√
Is OR delivering the goods? The journey to success in low-income countries		√	
The 2012 world health report “no health without research”: the endpoint needs to go beyond publication outputs		√	
Why ethics is indispensable for good-quality operational research		√	
Applying the ICMJE authorship criteria to operational research in low-income countries: the need to engage programme managers and policy makers		√	
References for scientific papers. Why not standardise to one global style?	√		
The power of data: using routinely collected data to improve public health programmes and patient outcomes in LMIC		√	√
Does research make a difference to public health? Time for scientific journals to cross the Rubicon	√		
Open access for operational research publications from low- and middle-income countries: who pays?		√	
Calling on Europe to support operational research in low-income and middle income countries. Lancet Global Health	√		
Public Health Action for public health action	√		
Ebola, fragile health systems and tuberculosis care: a call for pre- emptive action and operational research		√	√
Building the capacity of public health programmes to become data rich, information rich and action rich		√	√
Neglected tropical diseases and the sustainable development goals: an urgent call for action from the front line			√
**TOTAL PAPERS**	**4**	**9**	**5**

LMIC, Low-middle income country; ICMJE, International Committee of Medical Journal Editors

* Three papers had an influence on OR capacity building within SORT-IT and on policy/practice nationally or internationally, hence the sum of the columns being 18 not 15

Box 1 provides examples of how some of the ‘call for action’ papers shaped the philosophy and implementation of OR capacity building within the SORT IT partnership; Box 2 provides examples of how policy and practice at a national and international level was influenced.

Box 1. **Examples of how some of the ‘call for action’ papers shaped the philosophy and implementation of operational research capacity building within the SORT IT partnership**

**Example 1**

***Title of paper:*** The published research paper: is it an important indicator of successful OR at programme level?

***The call for action:*** A call for the published paper to be used as a yardstick for monitoring and measuring the outputs of operational research (OR) in disease control programmes in low to middle income countries (LMICs).

***How this call for action was taken up within the SORT-IT partnership***

•  Helped the SORT IT partnership to develop the 80-80-80-80 targets for its SORT-IT courses wherein the third target was that 80% or more of submitted papers must be published within 18 months of submission.

•  Convinced donors that publications could be considered an independent indicator of OR capacity building programme.

**Example 2**

***Title of paper:*** Is OR delivering the goods? The journey to success in low-income countries

***The call for action:*** This paper emphasised the intricate relation between OR and policy and practice, highlighted why OR findings might fail to affect policy and practice, and provided solutions and a checklist to ensure OR achieves its main goal of meeting health needs and improving health outcomes. This was a ‘call to action’ to say that if the criteria proposed for measuring research to policy and practice are adopted, this is an opportunity to better ensure that OR successfully impacts policy and practice, and to provide an objective way of monitoring the latter.

***How this call for action was taken up within the SORT-IT partnership***

•  Led Médecins Sans Frontières (MSF) to look beyond publications on ways of monitoring research impact. A software programme to monitor research impact for all MSF research was developed by MSF-UK.

•  Led the Union to look beyond publications and to routinely track and report the impact of research publications from SORT IT courses.

•  Helped SORT IT to include a target wherein at least 80% of research papers are tracked 18 months after submission to assess their on impact on policy and practice change.

**Example 3**

***Title of paper:*** Applying the International Committee of Medical Journal Editors (ICMJE) authorship criteria to operational research in low-income countries: the need to engage programme managers and policy makers

***The call for action:*** This paper discussed the dissonance between applying the ICJME authorship criteria and the OR goal of translating research findings into policy and practice. The authors called for recognising the contributions of programme managers and policy makers to the OR process and suggested that this recognition be included in the application of the ICMJE guidelines for permitted authorship.

***How this call for action was taken up within the SORT-IT partnership***

•  The SORT IT partnership embraced the philosophy of early engagement with programme managers and decision makers and their inclusion in study authorship

•  In National SORT IT courses (Myanmar, Zimbabwe, Sierra Leone, Liberia), this was taken one step further and national programme staff were invited to participate and provide input during the SORT IT plenaries so that their views of the potential for policy and practice could be included.

•  The SORT IT partnership modified its reporting framework to donors and other stakeholders as follows: a) in its quarterly reporting framework, it reported on how many national programme officers and/or policy makers were listed as co-authors on papers (linked to a target of 65%); and b) in its annual reporting logical framework it reported on how many national government staff were included as co-authors on papers. This was assessed annually against a specified indicator.

•  The spin-off from the latter is that good relevant research has a good chance of influencing policy and practice – the achievement being 65 – 70%.

Box 2. **Examples of how some of the ‘call for action’ papers resulting from the SORT IT courses influenced national and international policy and practice**

**Example 1**

***Title of paper:*** Language in tuberculosis services: can we change to patient-centred terminology and stop the paradigm of blaming patients?

***The call for action:*** An appeal to the global tuberculosis (TB) community – particularly the global Stop TB Partnership - to lead discussions on changing the paradigm of detrimental language in TB services.

***How this call for action was taken up outside the SORT-IT partnership***

•  Led the STOP TB partnership to convene a meeting to revise the Monitoring and Evaluation framework and lexicon of language in TB.

•  Made a major contribution to the crafting of the WHO Guidelines on revised definitions of TB patients and treatment outcomes: “WHO: Definitions and Reporting Framework for Tuberculosis” – 2013 revision (updated December 2014).

•  The Union conference and Union journals (IJTLD and PHA) refer to the paper and state that non-judgemental language be used for abstracts, presentations and in all manuscripts (conference submissions and author guidelines).

**Example 2**

***Title of paper:*** Building the capacity of public health programmes to become data rich, information rich and action rich

***The call for action:*** This paper presented the complementary adage of using both operational research and data-to-policy training in a decision-analysis framework, for evidence-informed decision making in public health. It called for Ministers of Health (MoH) to demand that operational research (OR) and decision analysis are used to make their Ministries ‘data-rich, information-rich and action-rich’. In parallel, advocacy and educational efforts were called for, to support MoH to understand the importance of this research evidence and ensure that it is collected and used effectively to improve their health services and health systems.

***How this ‘call for action’ was taken up outside the SORT-IT partnership***

•  A salient example of how this call for action was taken up is from the National TB programme in Zimbabwe (Takarinda KC, Harries AD, Mutasa-Apollo T, *et al.* Trend analysis of tuberculosis case notifications with scale-up of antiretroviral therapy and roll-out of isoniazid preventive therapy (IPT) in Zimbabwe, 2000–2018. BMJ Open 2020; 10: e034721).

•  The “data rich” was routine aggregate national data on TB case notification, numbers (and proportions) of people living with HIV (PLHIV) in antiretroviral treatment (ART) care and numbers (and proportions) of these ever commenced on IPT.

•  The “information rich” was the demonstration a strong association between national ART coverage in PLHIV rising from <1% to 88% between 2004 and 2018, national IPT coverage in PLHIV rising from <1% to 33% between 2012 and 2018 and TB case notification rates dropping 66% from 510 to 173 cases per 100,000 during this whole time period.

•  The “action rich” was the impetus to Zimbabwe of i) continuing to expand the HIV test and treat strategy to reach the UNAIDS 90% ART coverage target and the more recent 95% ART coverage target and ii) expanding TB preventive therapy but going for the 12-week course of weekly rifapentine and isoniazid rather than six months of daily isoniazid.

**Example 3**

***Title of paper:*** Neglected tropical diseases and the sustainable development goals (SDGs): an urgent call for action from the front line

***The call for action:*** The international community has pledged through the SDGs to eliminate neglected tropical diseases (NTDs) by 2030. This paper was an urgent call for a change in mind-set to garner support and hasten progress towards achieving this fast-approaching target. The authors advocated for an empowering approach aimed at propelling political momentum, milestones and targets for accountability, new science in drug development and increased funding particularly from G20 countries.

***How this call for action was taken up outside the SORT-IT partnership***

•  WHO-NTD department has taken on board several of the recommendations which have been integrated in the NTD roadmap.

•  The paper and its call were highlighted on the "World Neglected Tropical Diseases Day" https://www.itg.be/E/Article/30-january--the-first-ever-world-ntd-day

## Discussion

This is the first time that viewpoint papers derived from SORT IT courses have been formally assessed. Just over a third were ‘calls for action’; the rest were descriptive in nature. LMIC authorship and HIC-LMIC collaboration on these papers was high, but LMIC-first authorship and female representation was low. Three fifths of the ‘call for action’ papers contributed to the evolution of OR capacity building within the SORT-IT partnership.

The study strengths were that i) all discussion articles produced by the SORT IT partnership over the last 10 years were included and ii) we used universally acceptable parameters to assess access to, and utilisation of the viewpoint articles. The main limitations of this study were that i) online data on views and downloads of articles were only available for 17% of the papers, ii) citation counts might have been underreported without access to subscription-based databases like Web of Science and Scopus. That said, Google Scholar has been shown to be one of the most comprehensive sources for citations across different subject areas ^11^, so we believe that the effect of this limitation is minimal; and iii) the influence on policy and practice was only assessed through self-assessment by the authors of the paper; there was no external objective validation of the results and as such the methodology is open to bias. This is an important limitation of this aspect of the perspective paper.

This study highlights several interesting findings. First, although more than two thirds of viewpoint papers had LMIC authorship, the proportion of lead and last authors from LMICs was relatively low. Since these papers deal with issues specific to LMICs, having first and last authors from LMICs with knowledge and experience of these issues should be encouraged. Although course participants (predominantly from LMICs) may have an abundance of knowledge and experience to share in discussions and debates, most are not trained in how to craft these ideas and reflections into a paper. Furthermore, these viewpoint papers are not a primary output expected of the SORT IT courses; they are a ‘secondary output’. Thus, the writing process is often led by experienced faculty members (who often enrich or generate ideas from discussions with course participants and formulate these into thoughts), many of whom are from HICs. Fortunately, these faculty members have extensive experience of working in LMICs and are aware of their contexts. However, there is clearly room to do better, to find ways to encourage more LMIC-lead authors – participants and facilitators. Going forward, efforts should be made to increase the number of LMIC lead-authors on viewpoint papers that are generated from SORT IT courses. Many of the individuals who have been trained in SORT IT courses go on to perform OR in the future ^4, 5, 8^, and those who have co-authored a viewpoint paper, could be encouraged to lead on a viewpoint paper back in their home country as they become more experienced in scientific writing.

Second, female representation on the papers was low. This may be because there were fewer female mentors and mentees as compared to males on the SORT-IT courses ^3, 6^, particularly experienced faculty members. This is corroborated by findings from a previous evaluation of SORT IT courses showing that, of those participants enrolled on SORT IT courses between 2009 and 2014 who went on to become facilitators on subsequent courses, only 37% were female ^6^. As the authors of this evaluation pointed out, socio-cultural factors may also explain this observation - anecdotal evidence suggests that female facilitators experience difficulties in leaving home and travelling to distant course locations. One way to boost female involvement in paper writing is through the SORT IT alumni network; the latter provides an opportunity to actively encourage female SORT IT alumni to become OR fellows and to become drivers of OR.

Third, most of the journals in which viewpoints were published were open access which we recognise as being particularly important if we want to optimise readership in LMICs and ultimately influence policy and practice. Zachariah *et al.* ^6^, in their evaluation of 20 SORT IT courses conducted between 2009 and 2014, showed that article views/downloads for immediate open access articles were double those of closed access journals. We were unable to assess this correlation, as views and downloads of the viewpoint papers were only available for just under a fifth of the papers. Journals could clearly do a better job of presenting altmetric data of views, downloads, and citations of papers available on their websites. Such metrics are important and should be shared publicly as standard practice on journal websites. In our study, citation counts were used as proxy for utilisation. Establishing the relative value of a single citation count, however, is difficult in the absence of some sort of reference measure. A software tool that many online publishing platforms currently utilise (and which was available for two thirds of our papers) is Dimensions - a linked research data platform ^12^. Dimensions provides various metrics, one of which allows the citation performance of an article relative to similarly aged articles in its subject area to be compared. We cannot vouch for the validity of such a tool (this would need to be further explored), but metrics like this appear to be complimentary to citation counts and may provide a more nuanced overview of the utilisation of papers. 

Fourth, about three quarters of our viewpoints had an influence within or outside SORT IT and this compares favourably with the 55–66% change in policy and practice reported from previous SORT IT evaluations ^7, 9^. However, if we focus on changes in policy and practice outside the SORT IT partnership, this occurred in only a third of our ‘call for action’ papers - in other words, they had less influence in the wider realm than other (original research) SORT IT publications. This is probably because the papers in question are opinion pieces. One opinion paper may not be enough (policy and practice is influenced by all sorts of different inputs) and may not wield the same weight as an original research study when it comes to changing national or global thinking.

Finally, reflecting on the types of viewpoints that are being generated during SORT IT courses (one third being calls for action; two thirds being descriptive) should make us consider the added value of these types of papers. Our ‘call for action’ papers are meant to catalyse change and given the rich diversity and experience of people attending SORT IT courses this initiative should be championed. In addition to monitoring the OR outputs of our SORT IT courses, assessing viewpoint papers appears to be useful and encourages us to systematically monitor whether people we train in SORT IT go on to write viewpoint and perspective papers as they reflect a higher level of scientific thinking and critical reflection. Viewpoints contribute to challenging “more of the same” in health care and to “do things differently”.

In conclusion, viewpoint articles generated during SORT IT courses appear to complement original OR studies and are valuable contributors to the dissemination of OR practices in LMICs.

## Data availability

### Underlying data

DRYAD: Characteristics, utilization and influence of viewpoint articles from the Structured Operational Research and Training Initiative (SORT IT) – 2009–2020. https://doi.org/10.5061/dryad.fj6q573sk ^13^

This study contains the following underlying data:

- SORT IT_publications_viewpoint_articles_2010-2020.xlsx (SORT IT viewpoint article publications)
- Viewpoint_data.xlsx (article-based metrics of SORT IT viewpoint articles)

Data are available under the terms of the Creative Commons Zero "No rights reserved" data waiver (CC0 1.0 Public domain dedication).
