# Supplementary material for: Characteristics, utilisation and influence of viewpoint articles from the Structured Operational Research and Training Initiative (SORT IT) – 2009-2020
Source: F1000Res. 2021 Mar 10;10:198. [Version 1] doi: 10.12688/f1000research.27349.1 (PMC8218027; doi:10.12688/f1000research.27349.1)
Supplement: Peer review history

# Peer review: Reviewer response for version 1

**DOI:** 10.5256/f1000research.30226.r87937

**Published:** 2021-06-21

**Authors:** Prajjwal Pyakurel

**Affiliations:** 1 School of Public Health and Community Medicine, B.P. Koirala Institute of Health Sciences, Dharan, Nepal

**Title:**

- In the title, utilisation and the influences may carry the same meaning in minutely analyzed. So I think we can avoid overcrowding of words,

**Background: **

- In line number 9, what could be the indicators for improving program performance (in terms of ...??)?
- In the subheading,  SORT IT course and application we should also mention the follow up period after submission in peer reviewed journal.
-  Monitored and evaluated the characteristics and outputs of the OR courses. Can we add few examples?

**Results:**

- Influence on OR capacity building and on policy and/or practice.
- I am not clear about the use of the terminology in some form of change. I think we should be specific. 

**Discussion:**

- Although course participants (predominantly from LMICs) may have an abundance of knowledge and experience to share in discussions and debates, most are not trained in how to craft these ideas and reflections into a paper.  
- Comments: I also believe that besides training English language skills is crucial in writing the paper and non-native English speaker finds it difficult in writing the paper compared to native English Speaker and these things also should be highlighted.
- I believe a virtual platform could be another way besides SORT IT alumni network to involve female participants which can be added in the discussion.

**Conclusion:**

- I think we should also add policy and practice implications required further in depth assessment with robust methodology in the conclusion part.

Is the work clearly and accurately presented and does it cite the current literature?

Yes

If applicable, is the statistical analysis and its interpretation appropriate?

Yes

Are all the source data underlying the results available to ensure full reproducibility?

Yes

Is the study design appropriate and is the work technically sound?

Yes

Are the conclusions drawn adequately supported by the results?

Yes

Are sufficient details of methods and analysis provided to allow replication by others?

Yes

Reviewer Expertise:

Non-Communicable Diseases

I confirm that I have read this submission and believe that I have an appropriate level of expertise to confirm that it is of an acceptable scientific standard.

# Peer review: Reviewer response for version 1

**DOI:** 10.5256/f1000research.30226.r81281

**Published:** 2021-04-06

**Authors:** Desalegn Woldeyohannes

**Affiliations:** 1 Aklilu Lemma Institute of Pathobiology, College of Health Science, Addis Ababa University, Addis Ababa, Ethiopia

The paper is well written, concise, and clear with the study strengths and limitations clearly outlined. It is also interesting to read how some of the "view point"/"call for action’" papers shaped the operational research capacity building initiative as well as influence health policy and practice as presented in Box 1 and 2, respectively.

**Few comment/clarifications:**

- **Background section: **Inconsistent use of the conjunctions such as, "and or" (page 3, paragraph three last line) vs. "and/or" on the same page, paragraph 4 second from last line. 
- **Methodology section:** Inconsistency of using "international/national" vs. "international or national", page 4, first up to third lines; and again unnecessary space tap appeared between the two words.

Is the work clearly and accurately presented and does it cite the current literature?

Yes

If applicable, is the statistical analysis and its interpretation appropriate?

Yes

Are all the source data underlying the results available to ensure full reproducibility?

Yes

Is the study design appropriate and is the work technically sound?

Yes

Are the conclusions drawn adequately supported by the results?

Yes

Are sufficient details of methods and analysis provided to allow replication by others?

Yes

Reviewer Expertise:

Life Science, Public Health

I confirm that I have read this submission and believe that I have an appropriate level of expertise to confirm that it is of an acceptable scientific standard.
